# Remodeling tumor immune microenvironment (TIME) for glioma therapy using multi-targeting liposomal codelivery

**DOI:** 10.1136/jitc-2019-000207

**Published:** 2020-08-17

**Authors:** Zening Zheng, Jiaxin Zhang, Jizong Jiang, Yang He, Wenyuan Zhang, Xiaopeng Mo, Xuejia Kang, Qin Xu, Bing Wang, Yongzhuo Huang

**Affiliations:** 1 Artemisinin Research Center, Guangzhou University of Chinese Medicine, Guangzhou, China; 2 State Key Laboratory of Drug Research, Shanghai Institute of Materia Medica Chinese Academy of Sciences, Shanghai, China; 3 Shanghai University of Traditional Chinese Medicine School of Pharmacy, Shanghai, China; 4 NMPA Key Laboratory for Quality Research and Evaluation of PharmaceuticalExcipients, Shanghai, China; 5 Zhongshan Branch, the Institute of Drug Research and Development, ChineseAcademy of Sciences, Zhongshan, China

**Keywords:** tumor microenvironment, macrophages, brain neoplasms, immunotherapy

## Abstract

**Background:**

Glioblastoma (GBM) treatment is undermined by the suppressive tumor immune microenvironment (TIME). Seek for effective methods for brain TIME modulation is a pressing need. However, there are two major challenges against achieving the goal: first, to screen the effective drugs with TIME-remodeling functions and, second, to develop a brain targeting system for delivering the drugs.

**Methods:**

In this study, an α7 nicotinic acetylcholine receptors (nAChRs)-binding peptide ^D^CDX was used to modify the codelivery liposomes to achieve a ‘three-birds-one-stone’ delivery strategy, that is, multi-targeting the glioma vessel endothelium, glioma cells, and tumor-associated macrophages that all overexpressed α7 nAChRs. A brain-targeted liposomal honokiol and disulfiram/copper codelivery system (CDX-LIPO) was developed for combination therapy via regulating mTOR (mammalian target of rapamycin) pathway for remodeling tumor metabolism and TIME. Honokiol can yield a synergistic effect with disulfiram/copper for anti-GBM.

**Results:**

It was demonstrated that CDX-LIPO remarkably triggered tumor cell autophagy and induced immunogenic cell death, and meanwhile, activated the tumor-infiltrating macrophage and dendritic cells, and primed T and NK (natural killer) cells, resulting in antitumor immunity and tumor regression. Moreover, CDX-LIPO promoted M1-macrophage polarization and facilitated mTOR-mediated reprogramming of glucose metabolism in glioma.

**Conclusion:**

This study developed a potential combinatory therapeutic strategy by regulation of TIME and a ‘three-birds-one-stone’-like glioma-targeting drug delivery system.

## Introduction

Glioblastoma (GBM) is the most common malignant brain tumor, with a median survival of less than 15 months.^1^ There are two formidable challenges against the effective treatment of GBM. First, the access of most drugs to the brain is restricted by the blood-brain barrier (BBB), and thus there are very limited drugs available for GBM treatment. Second, GBM therapy is also hampered by the suppressive tumor immune microenvironment (TIME),^2^ which contains various types of non-cancerous cells including macrophages, dendritic cells (DCs), myeloid-derived suppressor cells (MDSCs), and T lymphocytes.^3^ As a case in point, tumor-associated macrophages (TAM) can comprise up to 50% of the tumor mass in malignant glioma and play an essential role in inducing drug resistance and metastasis.^4^ The analysis of the patients’ glioma tissues showed that the high tumor grade was linked to the enhanced amounts of the pro-tumorigenic TAM that created an immunosuppressive TIME.^4 5^ Of note, the primary population of TAM is the pro-tumor M2 subtype (TAM2). The repolarization from TAM2 to TAM1 is a promising anticancer therapeutic method.

Owing to hypoxia, GBM is characterized by the increased aerobic glycolysis (Warburg effect) that provides a main nutrient and energy source for the fast-proliferating cancer cells,^6^ and promotes the production of lactic acid that is a major immune suppressor in TIME.

Therefore, to develop a BBB-permeable therapeutic system that can remodel the TIME is an urgent need for GBM treatment. mTOR (mammalian target of rapamycin) signaling pathway is closely associated with the modulation of TIME by regulating multiple key physiological processes such as protein synthesis, autophagy, angiogenesis, and metabolism.^7^ mTOR belongs to the PI3K-related kinase family and its inhibition as an anticancer strategy has attracted great attention. In fact, metabolic reprogramming in GBM is a consequence of the PI3K/Akt/mTOR signaling.^8^ mTOR is a central pathway regulating the tumor microenvironment and its activation promotes GBM growth.^9^

In this study, we proposed a therapeutic strategy of targeting mTOR for remodeling the tumor metabolism and TIME via a BBB-penetrating liposomal system for codelivery of honokiol (HNK) and disulfiram/copper (DSF/Cu) complex. HNK is a main active compound in the Chinese herb Hou-Pu (bark of *Magnolia officinalis Rehd.et Wils*.). HNK has been demonstrated with antitumor activity; to our interest, it can inhibit the PI3K/mTOR pathway and promote anticancer immunity.^10^ Recently, mTOR signaling pathway has been revealed to regulate TAM modulation, autophagy, and tumor glycolysis,^7 11^ which are related to TIME. Therefore, we hypothesized that inhibiting mTOR pathway would be able to activate TIME. DSF, a clinically used anti-alcoholism drug, has been well-documented with potent anticancer activity in a form of complexation with copper ion (Cu) via a mechanism of crippling valosin-containing protein/p97 segregase adaptor NPL4.^12^ The anti-GBM effect of DSF/Cu was also investigated in several clinical studies.^13–15^ Therefore, the combination of HNK and DSF/Cu was proposed in this study for treating GBM. However, a challenge for this treatment is the poor BBB penetration of both drugs.

For brain drug delivery, receptor-mediated transcytosis is an important mechanism. For example, the nutrient transporters (eg, albumin-binding proteins) and the signaling transduction receptors on the cell surface (eg, integrin, LRP-1) are often used in brain delivery design. Nicotinic acetylcholine receptors (nAChRs) are highly expressed on the brain tumor capillary endothelial cells and glioma cells.^16–18^ Therefore, nAChRs are a useful target to mediate BBB-penetrating delivery of anti-GBM drugs.^19 20^ The α7 nAChRs-binding peptide ^D^CDX (GREIRTGRAERWSEKF, D-form sequence) has been demonstrated as a promising targeting ligand for brain delivery in glioma therapy.^21^ In this work, we applied ^D^CDX as a ligand for targeting TAM2 that also overexpressed α7 nAChRs and proposed a delivery strategy of ‘three-birds-one-stone’ for multi-targeting glioma capillary endothelium, glioma cells, and TAM2.

Herein, the HNK and DSF/Cu codelivery liposome system modified with ^D^CDX peptide was designed to treat GBM via regulating mTOR pathway for remodeling tumor metabolism and TIME.

## Results

### Characterization of CDX-liposomes

**Figure 1:**
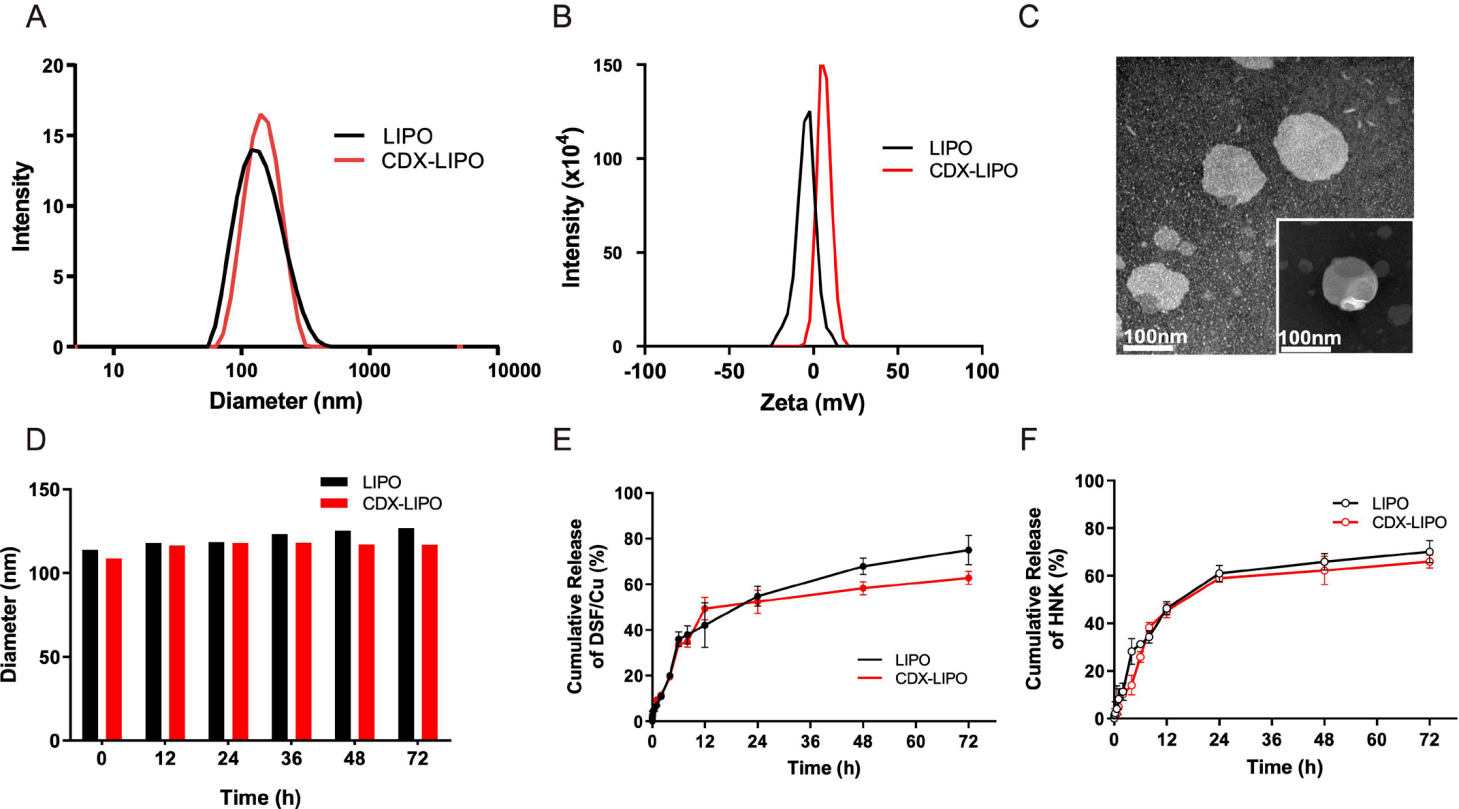
Characterization of the liposomes. (A) Particle size of CDX-LIPO (122.5 nm) and LIPO (118.7 nm). (B) Zeta potential of CDX-LIPO (1.36 mV) and LIPO (−6.17 mV). (C) TEM images of CDX-LIPO. (D) Stability of LIPO and CDX-LIPO in PBS containing 50% FBS. (E and F) In vitro release profiles of DSF/Cu (E) and HNK (F) from LIPO and CDX-LIPO in PBS. Data represents mean±SD (n=3). CDX-LIPO, CDX-modified liposomes; DSF/Cu, disulfiram/copper ion; FBS, fetal bovine serum; HNK, honokiol; LIPO, non-modified liposomes; PBS, phosphate-buffered saline; TEM, transmission electron microscope.

^D^CDX-PEG_2000_-DSPE was synthesized and confirmed by MALDI-TOF-MS assay (online supplementary figure S1) and used as a targeting ligand to modify the liposomes. The combo drugs DSF/Cu and HNK were encapsulated into the CDX-modified liposomes (termed CDX-LIPO, and the non-modified liposomes termed LIPO). The size and zeta-potential of the CDX-LIPO showed 122.5 nm and 1.36 mV, respectively, while those of the LIPO were 118.7 nm and −6.17 mV, respectively (figure 1A–B, online supplementary table S1). The zeta potential difference between LIPO and CDX-LIPO attributed to the positively charged peptide ^D^CDX. The TEM (transmission electron microscope) images showed the spherical morphology of CDX-LIPO (figure 1C). Drug encapsulation efficiency in both liposomes was greater than 85% and the loading capacity was 3% to 4% (table 1). Both liposomes remained stable in phosphate-buffered saline (PBS) containing 50% fetal bovine serum (FBS) (figure 1D). The in vitro release profile was also evaluated. A sustained release pattern was observed in both LIPO and CDX-LIPO (figure 1E–F). The total release rate was greater than 60% in 72 hours. It should be noted that complete release is not always ready to achieve due to the binding between lipids and drugs and forming complexes.

10.1136/jitc-2019-000207.supp1 Supplementary data

**Table 1:** Drug encapsulation efficiency (EE%) and drug-loading capacity (DL%)

	LIPO	CDX-LIPO
HNK EE%	89.2%±2.54%	87.8%±3.09%
HNK DL%	3.98%±0.42%	3.25%±0.28%
DSF/Cu EE%	68.9%±3.17%	64.5%±1.26%
DSF/Cu DL%	3.40%±0.42%	4.03%±0.21%

CDX-LIPO, CDX-modified liposomes; DSF/Cu, disulfiram/copper ion; HNK, honokiol; LIPO, non-modified liposomes.

## Cellular uptake and tumor spheroid penetration of CDX-LIPO

**Figure 2:**
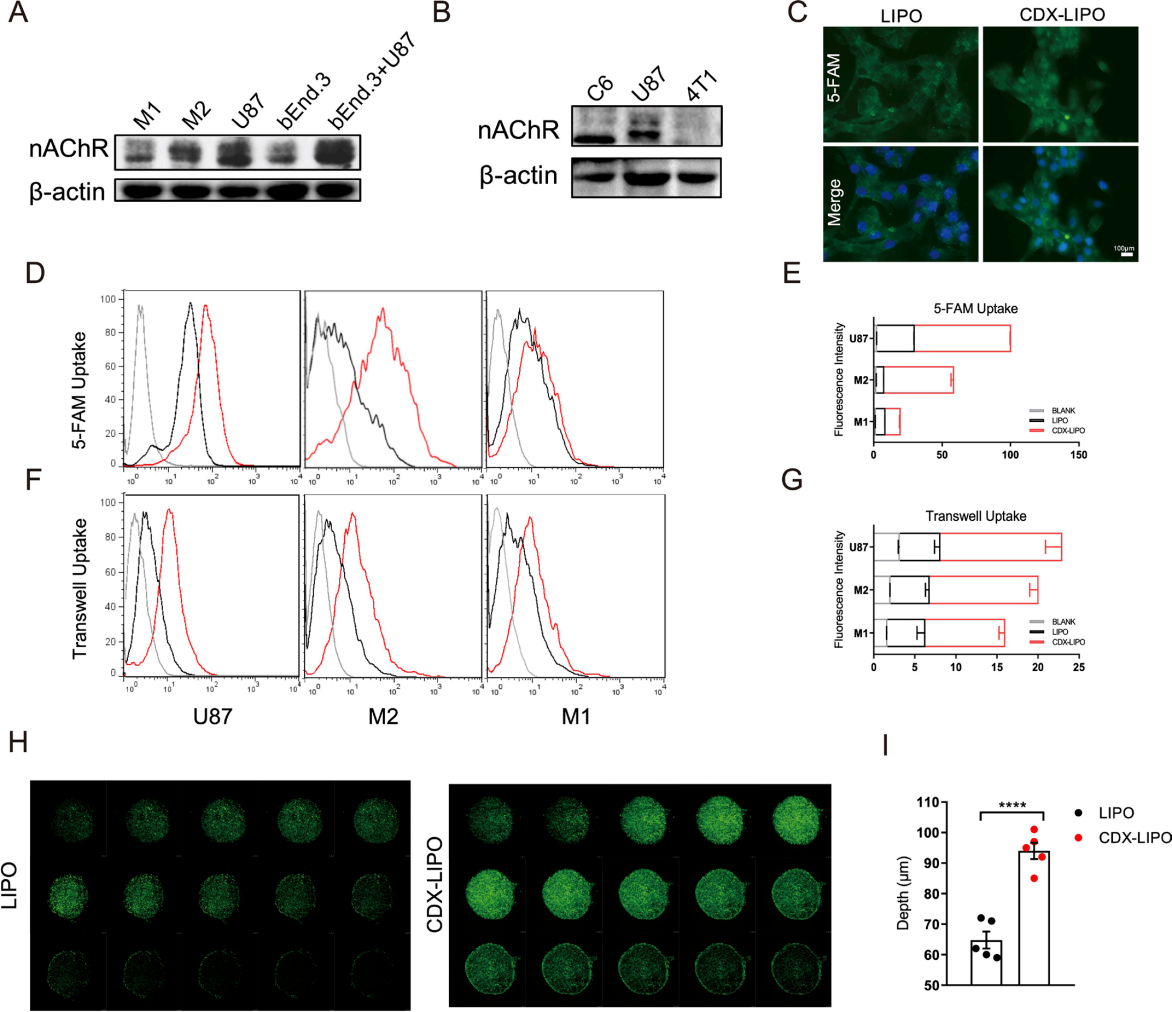
Cellular uptake and tumor spheroid penetration studies. (A and B) Expression of nAChRs in different cells. (C) Fluorescence images in U87 cells after incubation with the 5-FAM-labeled liposomes (scale bar: 100 µm). (D–G) Quantitative analysis of cellular uptake and transwell uptake efficiency in U87 cells and macrophages via flow cytometry. (H) In vitro spheroid penetration measurement by confocal microscope (200 µm for each depth level). (I) Quantitative analysis of penetration depth in spheroids. CDX-LIPO, CDX-modified liposomes; LIPO, non-modified liposomes; nAChR, nicotinic acetylcholine receptor.

A transwell co-culture model (bEnd.3/U87 cells) was used to investigate the transcytosis efficiency and evaluate the BBB penetration ability in vitro. The results showed that CDX-LIPO was efficient to penetrate through the bEnd.3 cell monolayer in the upper chamber and subsequently enter the U87 cells in the lower chamber (figure 2F–G). CDX-LIPO had a higher efficiency of endothelial monolayer penetration and intracellular delivery than LIPO, owing to the overexpressed nAChRs in the co-cultured bEnd.3 and U87 cells.

Moreover, the intratumor infiltration was evaluated using the cultured tumor spheroids. As expected, CDX-LIPO deeply penetrated the tumor spheroids whereas LIPO largely remained on the periphery of the spheroids (figure 2H–I).

### Cytotoxicity in glioma cells

The glioma cells were sensitive to DSF/Cu (IC_50_: 0.56 and 0.27 µg/mL for U87 and C6 cells, respectively), but HNK alone only showed moderate cytotoxicity (IC_50_: 20.1 and 13.3 µg/mL for U87 and C6 cells, respectively). The combination index analysis demonstrated a synergistic effect of DSF/Cu and HNK at 1:10 w/w with CI value of 0.72 to 0.74 (online supplementary table S2).

**Figure 3:**
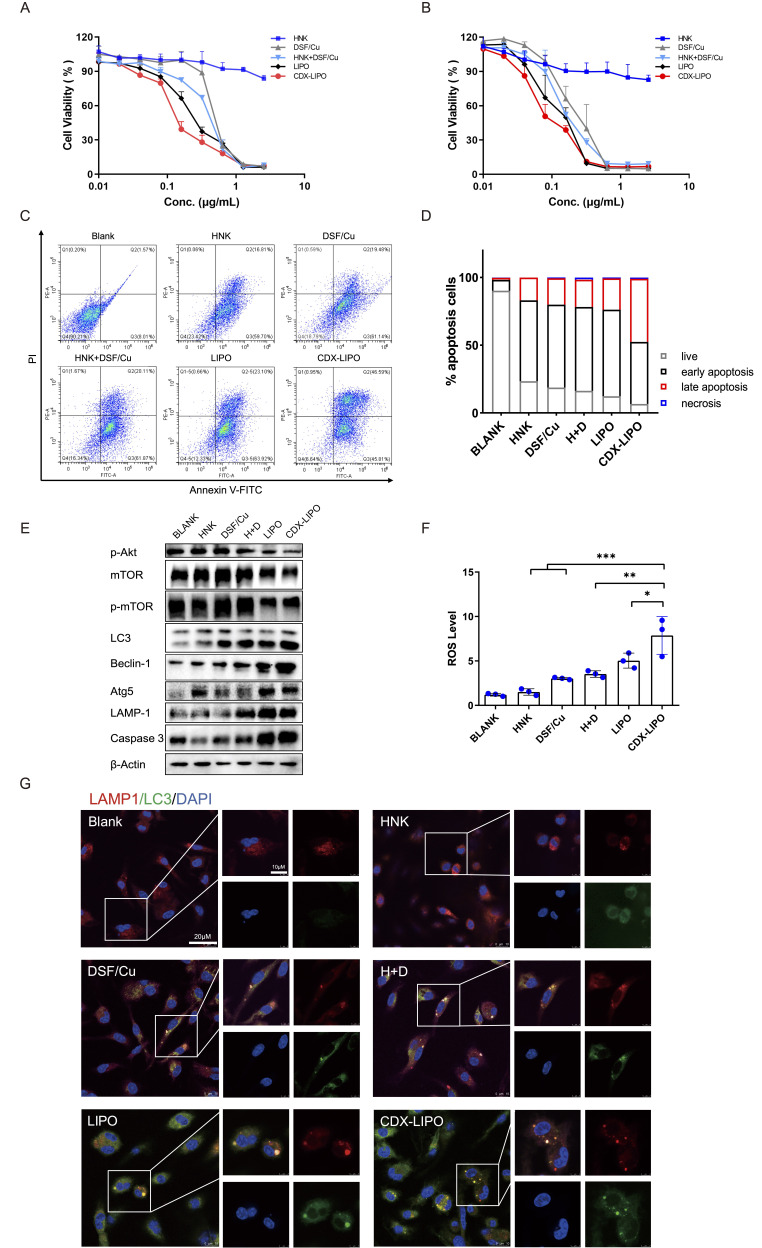
In vitro antitumor effect. (A and B) MTT assay in U87 cells and C6 cells. (C) Apoptosis assay in U87 cells via flow cytometry. (D) Statistical analysis of the percentage of apoptotic U87 cells. (E) The change of the autophagy biomarkers after treatment. (F) ROS production was enhanced after treatment. (G) Immunofluorescence analysis of intracellular autophagosome formation. CDX-LIPO, CDX-modified liposomes; DSF/Cu, disulfiram/copper ion; HNK, honokiol; LIPO, non-modified liposomes; mTOR, mammalian target of rapamycin; ROS, reactive oxygen species.

The IC_50_ values of CDX-LIPO and LIPO in U87 cells were 0.16 and 0.26 µg/mL, respectively (indicated by DSF/Cu concentration, figure 3A). CDX-LIPO showed the highest antitumor efficacy than LIPO and other groups of free-drug forms (IC_50_: DSF/Cu 0.56 µg/mL and the combo 0.38 µg/mL, whereas HNK showing minor cytotoxicity at the tested dose range). Besides, in C6 cells CDX-LIPO also exhibited increased cytotoxic effect compared with LIPO and free drugs (figure 3B). Online supplementary table S3 summarizes the IC_50_ values of different drugs. The apoptosis assay further confirmed the superiority of CDX-LIPO in killing the glioma cells (figure 3C–D).

The mechanisms of cell death induced by the liposomes were explored. Autophagy is characterized by a conversion of LC3I to LC3II and the upregulation of autophagy-related genes (Atg) family and autophagy-related proteins.^22 23^ Figure 3E shows that the phosphorylated Akt/mTOR was inhibited by CDX-LIPO treatment; the blockage of this signaling axis plays an important role in suppressing cancer growth.^24^ It is known that mTORC1 inhibition increases autophagy, whereas stimulation of mTORC1 reduces this process.^25^ Accordingly, CDX-LIPO markedly induced autophagy and cell death, evidenced by the upregulation of LC3-II, Atg5, LAMP1, and Beclin-1, which are the autophagic proteins.^26^ The increased autophagosome formation in the CDX-LIPO group was confirmed by confocal microscopic imaging (figure 3G). In line with the increased lysosome quantity, the LC3-positive phagosomes were found at a high level in the CDX-LIPO group but lower in the control group. Meanwhile, the flow cytometry results exhibited that the CDX-LIPO group greatly enhanced the production of ROS (figure 3F), which is a major inducer of autophagy.^27^ As a result, the apoptotic effector caspase 3 was upregulated (figure 3E), thus causing increased cell death. The results suggested that the anti-glioma effect was associated with autophagy and mTOR signaling.

### The immune-stimulation of CDX-LIPO

#### Immunogenic cell death induced by CDX-LIPO

**Figure 4:**
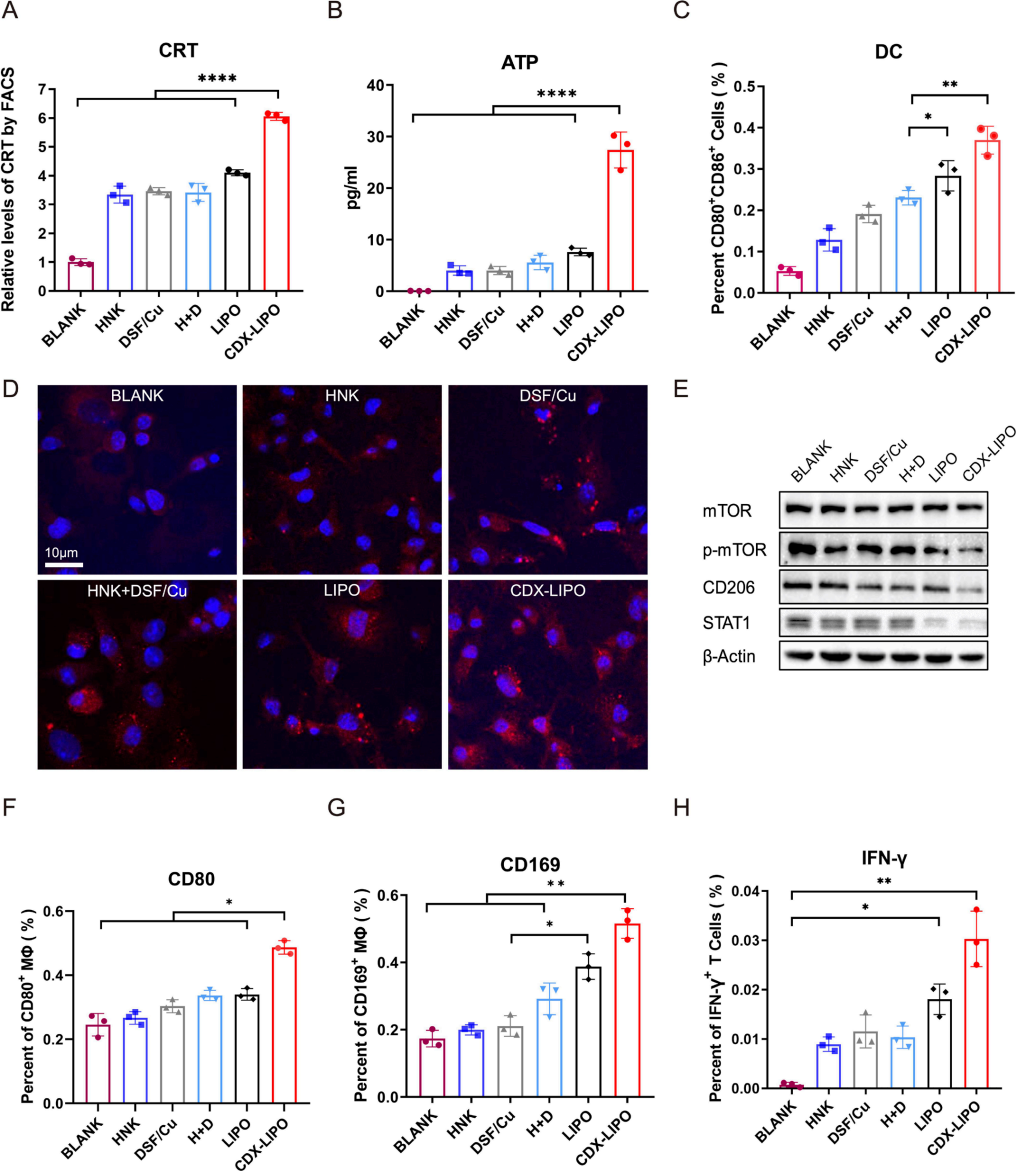
In vitro ICD induced by treatments and the effect on the antigen-presenting cells. (A) Flow cytometric quantification of calreticulin (CRT). (B) Extracellular adenosine triphosphate (ATP) level. (C) Flow cytometric analysis of dendritic cell (DC) maturation. (D) Fluorescent images of the exposed CRT on the C6 cell surface after treatments (scale bar: 10 µm). (E) M2 repolarization after treatments. (F and G) Activation of macrophages, as reflected by the biomarkers of CD80 and CD169. (H) Proportion of IFN-γ^+^ T cells primed with the matured DC cells that were activated by the drug-treated tumor cells. ATP, adenosine triphosphate; CDX-LIPO, CDX-modified liposomes; CRT, calreticulin; DC, dendritic cell; DSF/Cu, disulfiram/copper ion; HNK, honokiol; ICD, immunogeniccell death; IFN-γ, interferon gamma; LIPO, non-modified liposomes; mTOR, mammalian target of rapamycin.

Immunogenic cell death (ICD) is a kind of cell death releasing tumor antigens and eliciting immune responses, which is a useful strategy for activating tumor immunity and remodeling TIME. Of note, autophagy affects the characteristics of the dying cells by regulating the release of antigenic factors and thus can manipulate the immunogenicity of dying cells.^28^ Therefore, we examined the ICD effect in the glioma cells treated with CDX-LIPO by detecting the characteristic mediators (eg, calreticulin (CRT) and adenosine triphosphate (ATP)). The results revealed the efficient induction of ICD, as demonstrated by the increased CRT and ATP levels after CDX-LIPO treatment (figure 4A–B and D).

In accordance with an enhanced ICD effect, the CDX-LIPO-pretreated glioma cells promoted maturation of DCs (MHCII^+^CD86^+^) and activation of macrophages (CD169^+^/CD80^+^) (figure 4C, F and G). The matured DCs subsequently stimulated the interferon gamma (IFN-γ) secretion of the CD8^+^ T cells (figure 4H). The antigen-presenting cells play a vital role in initiating an effective adaptive immune response by presenting tumor antigens to the T cells for inducing anticancer cellular immunity.

## Repolarization of TAM by CDX-LIPO

### Targeting delivery studies

**Figure 5:**
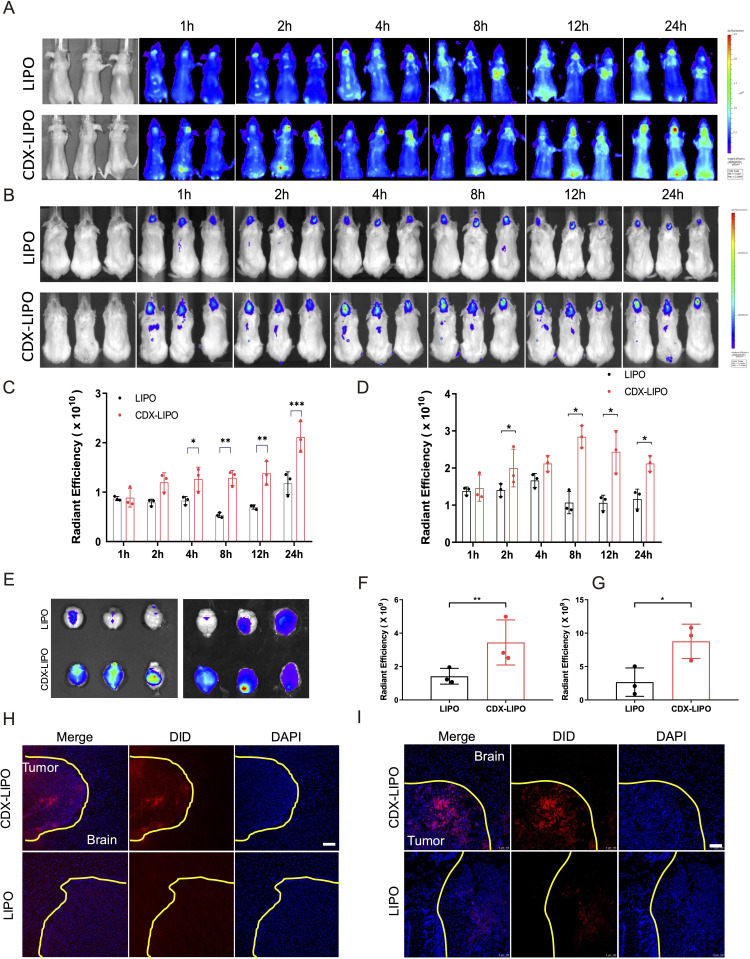
In vivo glioma-targeted delivery. (A and B) Biodistribution in the glioma-bearing mice (A: U87 nude mice; B: C6 Balb/c mice) after intravenous injection of the DiD-labeled liposomes. (C and D) Semi-quantitative ROI analysis of mean fluorescence intensity at the brain tumor sites (C: nude mice; D: Balb/c mice). Mean±SD, n=3. (E) Ex vivo images of the brains of the glioma bearing mice (left: nude mice; right: Balb/c mice). (F and G) Ex vivo radiant efficiency of the brains (F: nude mice; G: Balb/c mice). Mean±SD, n=3. (H and I) Immunofluorescence images of brain sections (H: nude mice; I: Balb/c mice) (scale bar: 100 µm). CDX-LIPO, CDX-modified liposomes; LIPO, non-modified liposomes.

The glioma-targeting delivery was evaluated in the mice bearing intracranial U87 or C6 tumors. The in vivo imaging showed that CDX-LIPO had a better targeting efficiency than LIPO, with higher brain retention and accumulation (figure 5A–D), owing to the overexpression of nAChRs in the glioma. The ex vivo imaging showed that the fluorescence signals of CDX-LIPO accumulated in the brain in both the glioma models (figure 5E–G). The immunofluorescence assay revealed that CDX-LIPO was widely distributed at the tumor site but barely detectable in the normal brain, while the LIPO group exhibited slight fluorescence in the glioma region (figure 5H–I). These results demonstrated that the CDX modification facilitated the liposomes to traverse the BBB and target the glioma microenvironment.

### In vivo anti-GBM studies in U87 and C6 orthotopic models

**Figure 6:**
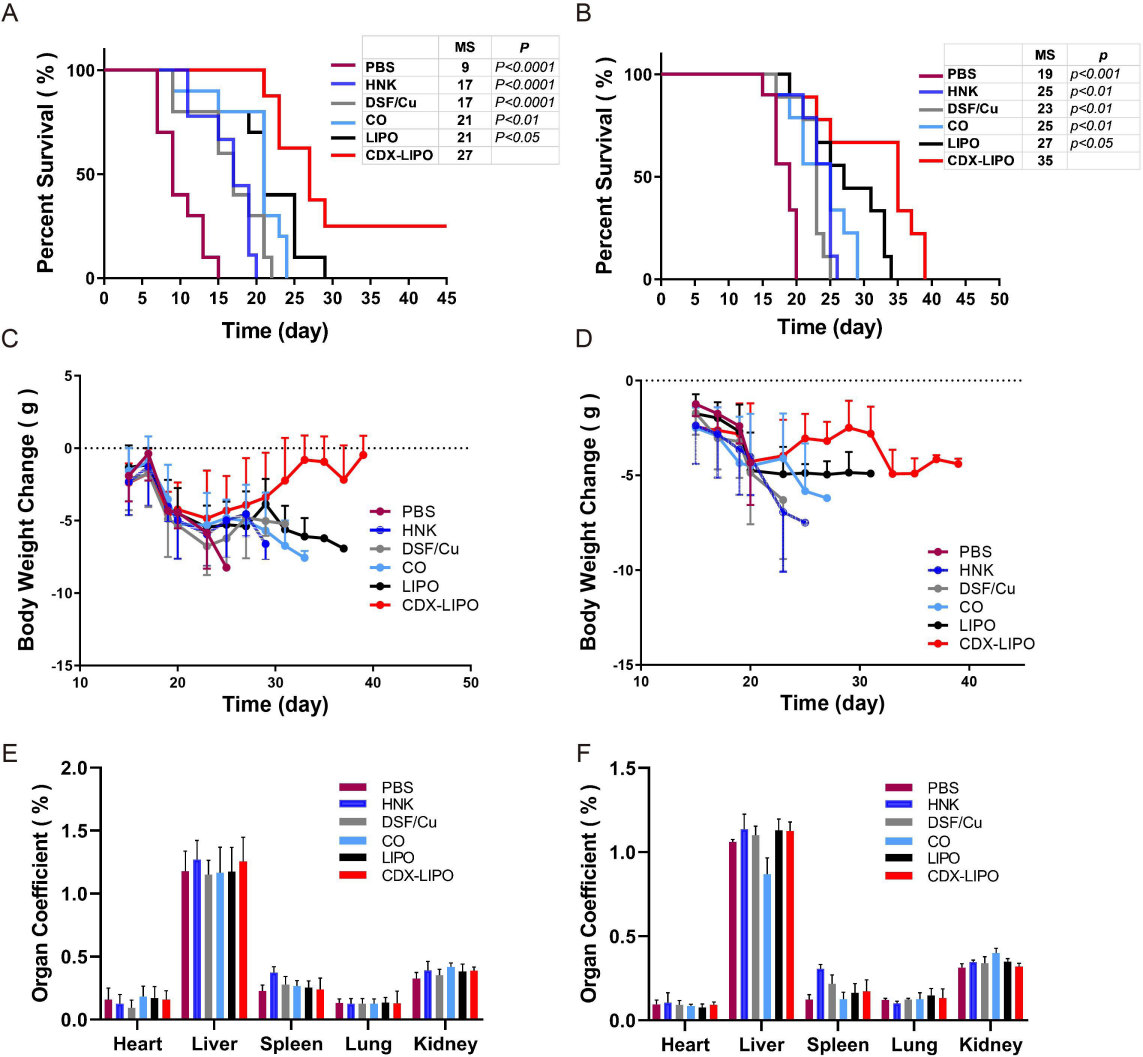
Anti-GBM efficacy on the orthotopic glioma models (left: C6 Balb/c mice; right: U87 nude mice). (A and B) Kaplan-Meier survival curves. P value indicates the comparison of median survival of the CDX-LIPO group versus other groups. (C and D) Body weight change. (E and F) Organ coefficients. Data are represented as means±SD (n=10). CDX-LIPO, CDX-modified liposomes; DSF/Cu, disulfiram/copper ion; GBM, glioblastoma; HNK, honokiol; LIPO, non-modified liposomes; PBS, phosphate-buffered saline.

The anti-GBM effect was demonstrated by the survival time of the orthotopic U87 and C6 glioma-bearing mice. C6 glioma as a commonly used animal brain tumor model and its immunological property was similar to human mesenchymal GBMs.^29^ As shown in figure 6A, the median survival time of the orthotopic C6 mice in the CDX-LIPO group was 27 days, which was significantly longer than that of the mice treated with PBS (9 days, p<0.0001), free-drug injections (17 days, p<0.0001), free-drug combo (21 days, p<0.01), and LIPO (21 days, p<0.05). There were 30% of the mice in the CDX-LIPO group still survived at the end of the experiment.

Moreover, the body-weight variation of mice was recorded. It showed that the body weight of all mice including the PBS control group declined, but there was no significant difference in the first 20 days among the groups (figure 6C, online supplementary table S4). The body-weight loss was due to the poor appetite caused by the brain-tumor burden, which was commonly seen in the orthotopic glioma models. The organ coefficient assay (figure 6E) and H&E histological examination (online supplementary figure S2) showed no obvious pathological changes in the major organs, implying that the biocompatibility of CDX-LIPO.

Moreover, the therapeutic strategy was further evaluated in another model (orthotopic U87 glioma in nude mice). The CDX-LIPO group exhibited the potent anti-GBM effect (figure 6B, D and F), with longer survival than other groups. Such superiority of CDX-LIPO could be attributed to the enhanced brain delivery.

### Anti-GBM mechanisms

**Figure 7:**
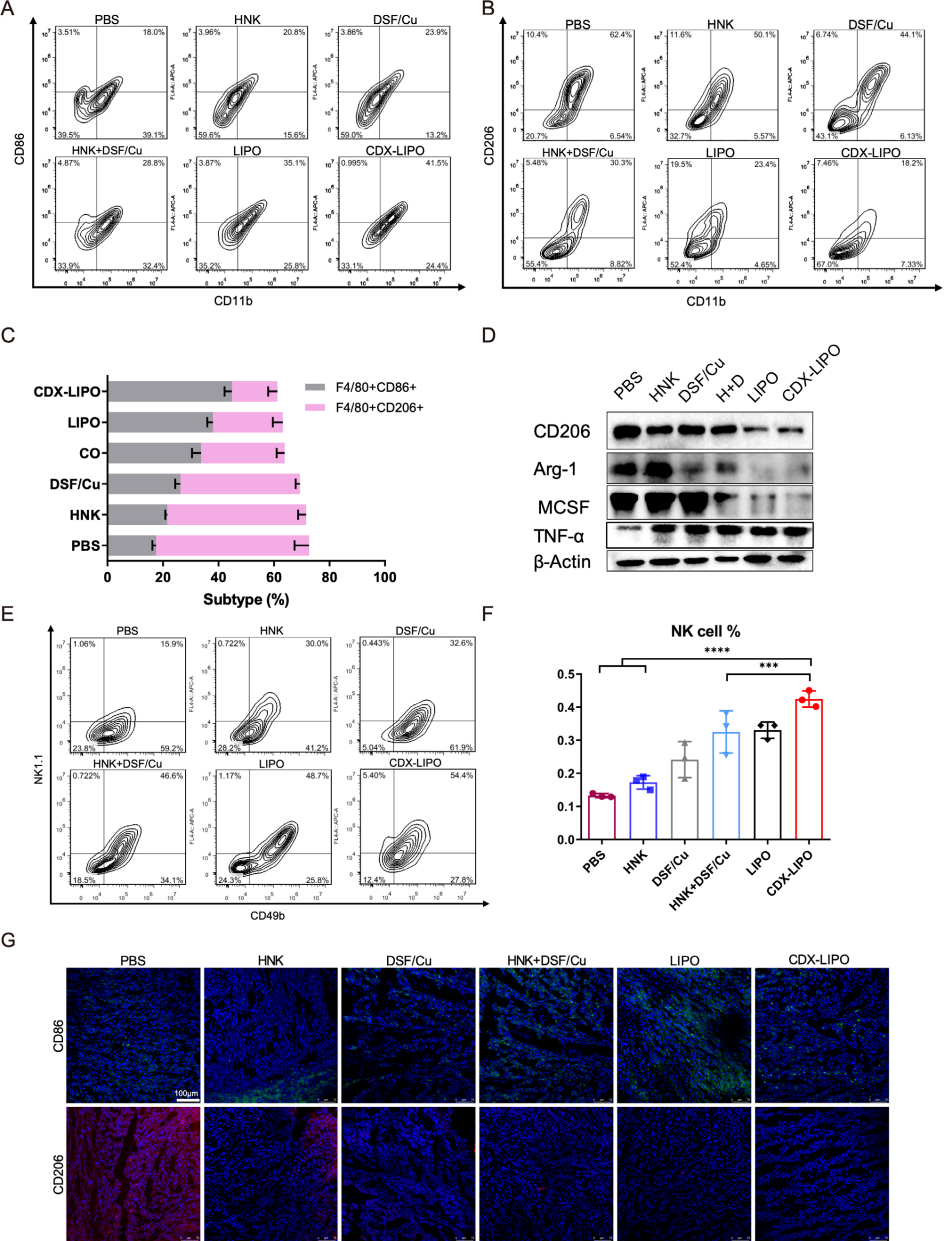
The intratumor TAM and NK cells in the U87-glioma nude mice after treatment. (A, B, C) Flow cytometric analysis of M1Φ and M2Φ in the brain tumors. (D) Repolarization of M2Φ. (E, F) Flow cytometric analysis of NK cells. (G) Immunofluorescence staining of M1Φ (green) and M2Φ (red) in the brain tumors (scale bar: 100 µm). CDX-LIPO, CDX-modified liposomes; DSF/Cu, disulfiram/copper ion; GBM, glioblastoma; HNK, honokiol; LIPO, non-modified liposomes; NK, natural killer; PBS, phosphate-buffered saline; TAM, tumor-associated macrophages; Φ, macrophages.

Immunosuppression is typically associated with brain tumors, and therefore, modulation of TIME is a potential anti-GBM strategy.^30 31^ In the U87-bearing nude mice, the innate immunity was examined after treatment. The TAM quantity in the glioma was measured. It showed the proportion of M1Φ in the CDX-LIPO group was increased (41.5%), significantly higher than that of other groups: PBS (18.0%), HNK (20.8%), DSF/Cu (23.9%), combo (26.8%), and LIPO (35.1%) (figure 7A–C), whereas the percentage of M2Φ was remarkably reduced after CDX-LIPO treatment. The immunofluorescence staining and western blotting results further confirmed the efficient repolarization from the protumor M2 to antitumor M1 subtype, reflected by the downregulation of the M2-associated factors (eg, Arg1, CD206, and MCSF) and the upregulation of the M1-associated CD86 and tumornecrosis factor-alpha (TNF-α) (figure 7D and G).

Of note, M2Φ inhibits the natural killer (NK) cells, and M1Φ primes the NK cell-mediated antitumor immunity.^32^ The NK cells play a critical role in innate immunity by expressing perforin and granzymes to induce apoptosis or necrosis of cancer cells.^33^ However, their antitumor functions are often impaired in advanced brain cancer patients.^34^ It was observed that the population of NK cells (NK1.1^+^CD49b^+^) in the tumor was significantly increased after treatment with CDX-LIPO (figure 7E and F).

**Figure 8:**
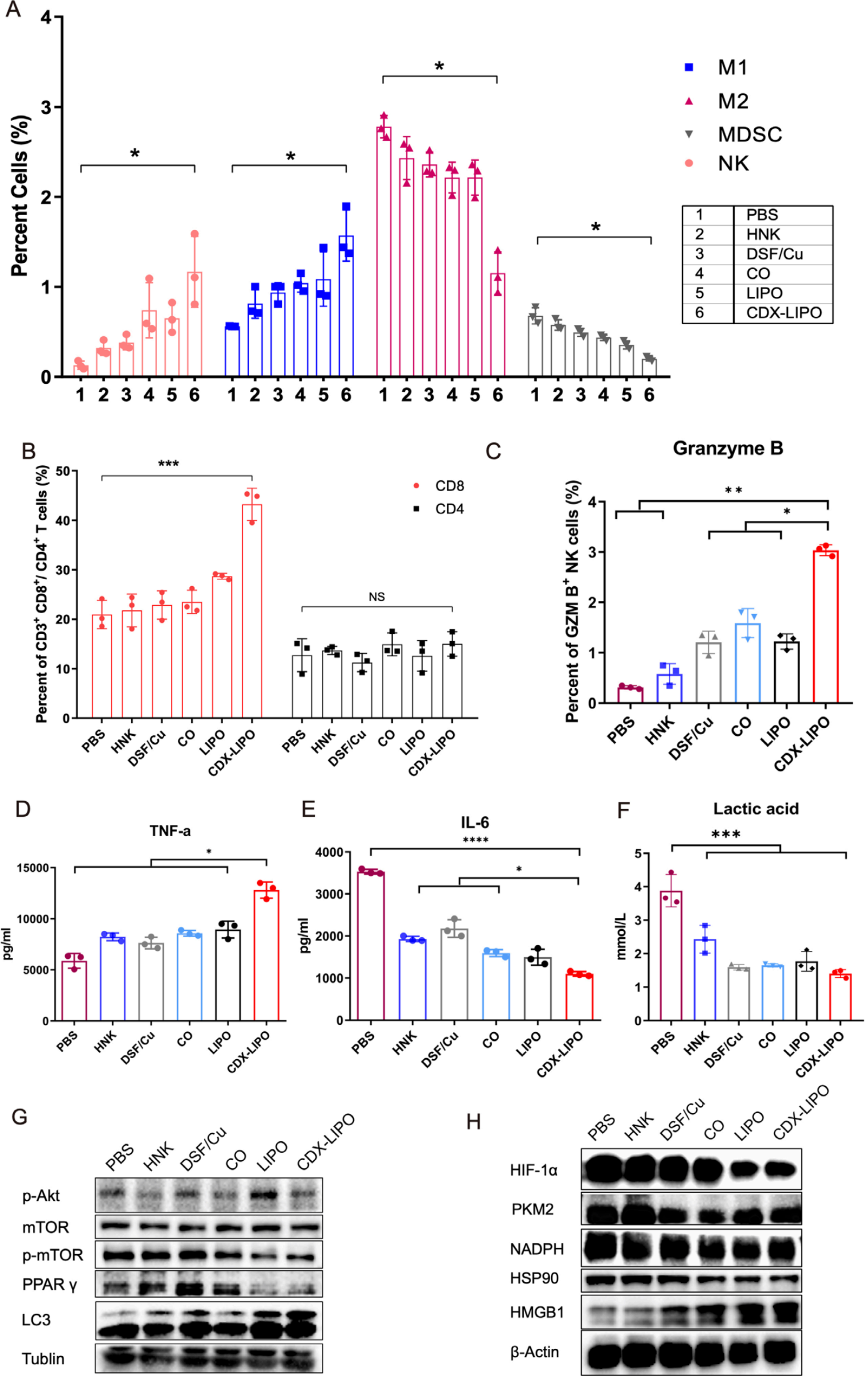
The activated TIME in the C6 glioma. (A) Flow cytometric analysis of MDSC, NK cells, M1Φ, and M2Φ in the brain tumors. (B) Flow cytometric analysis of T cells in the spleens. (C) Detection of granzyme B^+^ NK cells via FACS. (D, E) Intratumor TNF-α and IL-6 levels by ELISA assay. (F) CDX-LIPO reduced the production of intratumor lactic acid. (G, H) Western blot assay of mTOR and the related proteins in the brain tumors. CDX-LIPO, CDX-modified liposomes; DSF/Cu, disulfiram/copper ion; HNK, honokiol; IL-6, interleukin 6; LIPO, non-modified liposomes; MDSC, myeloid-derived suppressor cell; mTOR, mammalian target of rapamycin; NK, natural killer; PBS, phosphate-buffered saline; TIME, tumor immune microenvironment; TNF-α, tumor necrosis factor-alpha; Φ, macrophages.

Furthermore, both the innate and adaptive immunity were examined in the immunocompetent Balb/c mice bearing orthotopic C6 glioma. CDX-LIPO treatment elevated the percentage of M1Φ in the tumors, which was about 1.5-fold to 2-folds higher than other groups (figure 8A). Consistently, the amount of both the total NK cells and the granzyme B-positive NK cells was significantly increased by CDX-LIPO treatment (figure 8A and C). By contrast, the proportion of immunosuppressive M2Φ and MDSCs in the tumors of the CDX-LIPO group was lower than other groups (figure 8A).

The success of immunotherapy also relies on the activation of tumor-specific T cells.^35^ Our results revealed that there was a two-fold increase in the proportion of the splenic CD3^+^CD8^+^ cytotoxic T cells in the CDX-LIPO group, compared with other groups, whereas the CD3^+^CD4^+^ T helper cells showed no major difference among the groups (figure 8B).

Furthermore, the cytokines in the glioma were measured. It was found that the antitumor TNF-α was significantly enhanced by CDX-LIPO treatment, but the protumor interleukin 6 (IL-6) was downregulated (figure 8D–E).

The Akt/mTOR signaling pathway is associated with the regulation of glucose metabolism and M2 polarization.^36^ Lactic acid, a glycolysis product, and M2Φ consequently promoted the development of TIME. Figure 8F shows CDX-LIPO treatment reduced the production of lactic acid in the brain tumor. Lactic acid is a known immune suppressor in the tumor microenvironment, which represses both the antitumor innate and adaptive immunity.^37^ Our results also revealed that the key regulators of glucose metabolism, pyruvate kinase M2 (PKM2) isoform and HIF-1α, were downregulated in the glioma, as a result of inhibition of Akt/mTOR-signaling by CDX-LIPO (figure 8G). Accordingly, NADPH and HSP90, the glycolysis-associated factors,^38 39^ were suppressed (figure 8H). It demonstrated that mTOR-mediated cancer metabolism also played a role in remodeling the TIME. Our results were consistent with the knowledge that Akt/mTOR signaling pathway contributes to the Warburg effect.^36 40^

A tumor proliferation-associated protein PPAR was decreased, but an autophagy-related protein LC3-II and an ICD marker HGBM1 were upregulated (figure 8G and H). It demonstrated the suppression of tumor growth and enhanced autophagy and tumor immune responses.

In summary, the antitumor effect elicited by CDX-LIPO was via multiple mechanisms. In addition to the cytotoxic effect on tumor cells, CDX-LIPO also yielded anticancer immune responses. CDX-LIPO could act on mTOR signaling, induce autophagy and ICD, repolarize TAM, and inhibit the aerobic glycolysis (Warburg effect) as well, thus remodeling the TIME. The triggered antitumor immunity included the increased M1Φ, NK cells, and cytotoxic T cells, while the immunosuppressive M2Φ and MDSC were decreased. MDSC exhibits the local and systemic immunosuppression in the GBM patients,^41^ and the density of TAMs and MDSCs in the glioma inversely correlates with the patient survival rate.^3^

## Discussion

DSF is an old drug used in clinics for over six decades for preventing alcohol abuse, which has the well-demonstrated pharmacokinetic profiles and biosafety. Repurposing DSF as an anticancer agent has been considered with great clinical value.^12^ We previously demonstrated that DSF-based therapy could modulate TAM and trigger antitumor immunity.^31 42^ In this work, we first proposed a combination therapy of DSF/Cu and HNK; HNK is an antitumor natural compound. A BBB-penetrating and glioma-directing liposomal codelivery system modified with ^D^CDX peptide was developed for targeting α7 nAChRs that were overexpressed in the glioma vascular endothelium, glioma cells, and TAM2.

The mTOR pathway integrates spatiotemporal information of the tumor growth including apoptosis, autophagy, cell metabolism, and immune responses.^36^ For example, the mTOR signaling is an important pathway for regulating macrophage polarization and NK cells activity,^43 44^ primarily via the PI3K/Akt/mTOR axis.^45^ Therefore, it plays a central role in regulating tumor microenvironment. The CDX-LIPO was applied to target the mTOR pathway and remodel the TIME for anti-GBM treatment. Of note, mTOR plays an essential role in cancer metabolism; for example, mTOR signaling controls glucose metabolism by upregulating glycolytic enzymes and glucose transporters and activating the transcription factors (eg, HIF-1α).^36^ Our work demonstrated that the inhibition of Akt/mTOR signaling promoted autophagy and the repolarization of TAM (M2→M1), as well as repressed glucose metabolism and lactic acid production, thus regulating the populations of the immune cells and the expression of cytokines. In addition, the ICD effect induced by CDX-LIPO facilitated the release of the ‘find-me’ signals (eg, ATP) and the maturation of antigen-presenting cells (DCs and macrophages), and consequently activated cytotoxic T cells.

GBM immunotherapy has recently attracted great attention. Traditionally, the central nervous system was considered as ‘immune privilege’, but this perception has been changed by two recent important discoveries of the existence of a lymphatic system both in the murine and human brain.^46 47^ However, due to the BBB rejecting the access of most drugs, the progress of GBM immunotherapy has not met the expectation. For example, the first large randomized clinical trial (CheckMate 143) of anti-PD-1 therapy failed to extend the overall survival of the recurrent GBM patients, without reaching its primary endpoint.^48^ Our work developed a novel codelivery system that simultaneously targeted BBB, glioma cells, and TAM, thus providing a useful strategy for GBM immunotherapy. It proposed a useful mTOR-targeting therapeutic method for anti-GBM based on remodeling cancer metabolism and TIME.

## Conclusions

In summary, we developed a GBM-targeted liposomal codelivery system and discovered that α7 nAChRs can be used as a promising delivery target for achieving a ‘three-birds-one-stone’ strategy for action on glioma microenvironment. It revealed that CDX-LIPO can suppress GBM growth by targeting mTOR pathway, inducing autophagy and ICD, and regulating tumor metabolism and TIME. The remodeling TIME involved the reduced aerobic glycolysis and lactate production, repolarization of TAM, activation of DCs, NK cells, and cytotoxic T cells, and inhibition of MDSC. The secretion of antitumor TNF-α was increased, while the protumor IL-6 decreased. The findings suggested that CDX-LIPO was potential for anti-GBM immunotherapy, and the liposomal formulation offers a great promise for clinical translation.

## Experimental section

### Materials

Soybean phosphatidylcholine (SPC), cholesterol, DSPE-PEG_2000_, and DSPE-PEG_2000_-Mal were supplied by Shanghai Advanced Vehicle Technology Pharmaceutical Co, Ltd (Shanghai, China). Disulfiram (DSF), honokiol (HNK), and 1,1-Dioctadecyl-3,3,3,3-tetramethylindodicarbocyanine (DiD) were obtained from Dalian Meilun Biotechnology Co, Ltd (Dalian, China). FAM (5-carboxyfluorescein) was purchased from Sigma-Aldrich (St. Louis, USA). Copper (II) Chloride Dihydrate was obtained from Sinopharm Chemical Reagent Co, Ltd. (Shanghai, China). ^D^CDX peptide (sequence: CGREIRTGRAERWSEKF) was synthesized by Bankpeptide Biological Technology Co, Ltd (Hefei, China).

Macrophage colony-stimulating factor (M-CSF) and murine IFN-γ and IL-4 were purchased from PeproTech (Rocky Hill, USA), and lipopolysaccharide (LPS) from Sigma-Aldrich (St. Louis, USA). The primary antibodies of phospho-mTOR, phospho-Akt, LC3, Atg5, LAMP-1, Beclin-1, caspase3, STAT1, PPAR γ, and HSP90 were purchased from Cell Signaling Technology (Boston, USA). Collagenase IV, hyaluronidase, and the primary antibodies of β-actin and α-tubulin were purchased from Sigma-Aldrich (St. Louis, USA), and the primary antibodies of α7 nAChRs, mannose receptor (CD206), mTOR, Arg-1, MCSF, NADPH, HMGB1, PKM2, and TNF-α were purchased from Abcam (Cambridge, UK). Anti-HIF-1α was purchased from Novus (Shanghai, China). MS CD16/CD32 Pure 2.4G2, Cy5.5 Anti-mouse CD45, PE Anti-mouse NK-1.1, APC Anti-mouse CD49b, PE Anti-Mouse Ly-6G and Ly-6C, APC Anti-CD11b, APC Anti-mouse CD206, APC Anti-mouse CD86, PerCP/Cy5.5 Anti-mouse CD80, APC Anti-mouse CD8, FITC Anti-mouse CD4, PE Anti-mouse Granzyme B, PE-Cy7 Anti-mouse IFN-γ, and PE Anti-mouse F4/80 Antibodies were purchased from BD Biosciences (San Jose, USA). Horseradish peroxidase (HRP)-conjugated goat anti-rabbit/mouse lgG secondary antibody was purchased from Beyotime (Shanghai, China).

### Cell lines

U87 cells (human GBM cells), C6 cells (rat GBM cells), and bEnd.3 cells (murine brain endothelial cells) were provided by Shanghai Cell Bank of Chinese Academy of Sciences (Shanghai, China), and cultured in Dulbecco’s modified Eagle’s medium (DMEM) containing 10% FBS (Gibco) at 37°C in a humidified incubator containing 5% CO_2_.

### Animals

Female Balb/c nude mice (3 to 4 weeks) and Balb/c mice (4 to 5 weeks) were supplied by Shanghai SLAC Laboratory Animal Co, Ltd (Shanghai, China), and housed under specific pathogen-free conditions. All animal experiments were performed in accordance with the ethical regulations and approved by the Institutional Animal Care and Use Committee of Shanghai Institute of Materia Medica, CAS (IACUC No. SYXK2015-0027).

### Synthesis and characterization of materials

^D^CDX and DSPE-PEG_2000_-Mal were conjugated through a sulfhydryl-maleimide coupling method. In brief, DSPE-PEG_2000_-Mal in DMF was slowly added into the ^D^CDX peptide solution (PBS, pH 7.4). The purified materials were obtained by dialysis (MWCO 3.5 kDa) against distilled water. The product was confirmed by MALDI-TOF Mass.

The liposomes were prepared by a thin film-hydration method. SPC, cholesterol, DSPE-PEG_2000_, or ^D^CDX-PEG_2000_-DSPE (20/4/1, w/w/w) were dissolved in chloroform. DSF/Cu was prepared via a previous method,^42^ and DSF chelated with CuCl_2_ in ethanol solution at a molar ratio of 1:1. DSF/Cu and HNK (1:8, w/w) were dissolved in ethanol. The solutions of the lipids and the drugs (25:1, v/v) with a ratio of lipids-to-drugs of 25:9 (w/w) were mixed and then subjected to rotary evaporation to form a thin film in a round flask. The dry film was hydrated by 5% glucose, followed by ultrasonic treatment in water bath. The resultant liposomes were extruded through the 0.4 µm and 0.2 µm polycarbonate membranes using Avanti Mini Extruder (Avanti Polar Lipids, Alabaster, USA). The free drugs were removed using a Sephadex G-50 column eluted with saline. The resultant liposomes contained a combination dose (~1:10 w/w) of DSF/Cu and HNK. The fluorescent dye-labeled liposomes were prepared similarly by using FAM or DiD dye.

### Characterization of CDX-LIPO

The particle size and zeta potential of the liposomes suspended in distilled water were determined by a Malvern Zeta analyzer (Nano-ZS instrument, Malvern, UK) after dilution with water 1/10 (v/v). The temperature was kept at 25°C during measurement.

The morphology of CDX-LIPO was observed under a transmission electron microscope (TEM, 120 kV, FEI Tecnai G2 Spirit). CDX-LIPO was placed on a copper grid covered with nitrocellulose and negatively stained with phosphotungstic acid and dried at room temperature.

The concentrations of DSF/Cu and HNK were determined by a high-performance liquid chromatography (HPLC) instrument (Agilent 1200, USA) equipped with a Zorbax Eclipse C18 column (4.6 mm×150 mm, 5 µm) (Agilent Technologies, USA). The mobile phase for HNK assay was water and methanol (80:20) at a flow rate of 1 mL/min, with a detection wavelength of 294 nm. The mobile phase for DSF/Cu assay was acetonitrile and water (85:15) containing 0.1% TFA at a flow rate of 1 mL/min, with a detection wavelength of 254 nm. The column temperature was kept at 25°C. Drug loading capacity (DL%) and encapsulation efficiency (EE%) were calculated according to the following formula:

DL% = M_0_/M_t_×100%

EE% = W_0_/W_t_×100% where M_0_ was the total drug amount adding in preparation, M_t_ was the total weight of liposomes, W_0_ was the amount of drugs in liposomes, and W_t_ was the total amount of drugs added in preparation.

### Stability of the liposomes and in vitro drug release

The stability of the liposomes suspending in PBS (pH 7.4) containing 50% FBS was monitored by size change. The particle size of the samples was measured at different time points after dilution with ultrapure water 1/10 (v/v) by a dynamic light scattering method as described previously.^30^

The in vitro drug release measurement was performed using a dialysis method at a shaker (150 rpm, 37°C). In brief, LIPO or CDX-LIPO was filled in a dialysis tube (MWCO 10 to 12 kDa) with PBS (pH 7.4) as a release medium to satisfy the sink condition. At different time points, the samples were withdrawn for measurement and the same volume of PBS was supplemented. DSF/Cu and HNK released from the liposomes were analyzed by the HPLC method described above. The cumulative drug release was calculated.

### The polarization of M1Φ and M2Φ

The bone marrow-derived macrophages (BMDM) were collected from the Balb/c mice (male, 4 to 5 weeks) using a standard procedure. Briefly, the bone marrow was flushed with DMEM, and the cells were collected and cultured in DMEM containing 10% FBS and 20 ng/mL M-CSF for 5 days. They were treated with 100 ng/mL LPS and 20 ng/mL IFN-γ for M1 polarization, or 40 ng/mL IL-4 for M2 polarization, for 24 hours, according to a protocol previously reported.^42^

### Generation of mouse bone marrow-derived DCs

The bone marrow-derived DCs (BMDC) were collected from the Balb/c mice (male, 4 to 5 weeks, specific pathogen-free) femurs and cultured in the six-well plates with 20 ng/mL granulocyte M-CSF (PeproTech) and 20 ng/mL IL-4 (PeproTech). On day 3 and 5, the medium was half-renewed and then the non-adherent cells were collected for further experiments.

### Cellular uptake of FAM-liposomes

U87 cells, M1 cells, and M2 cells were seeded into the 12-well plates and cultured for 24 hours. The cells were incubated with the FAM-liposomes (5 µM) at 37°C for 2 hour. The cells were harvested; a portion of the cells was used for flow cytometry (BD FACS Calibur, USA) for quantitative analysis and the other cells were fixed with 4% paraformaldehyde and stained with DAPI for confocal fluorescence imaging (Carl Zeiss, Germany).

### Targeting delivery studies using in vitro BBB model

The in vitro BBB culture model was constructed according to a previous report.^42^ The bEnd.3 cells were seeded onto the transwell upper chambers (0.4 µm pore size) and the U87 cells were in the lower chambers (Corning, USA). The cell monolayer integrity was monitored by transendothelial electrical resistance (TEER). When the TEER was higher than 200 Ω·cm^2^, the FAM-liposomes (50 µM) were added to the culture medium in the upper chambers for 6-hour treatment. The U87 cells were then collected for flow cytometric assay (BD FACS Calibur, USA).

### Penetration of tumor spheroid

The U87 cells were seeded at a density of 5×10^3^ cells per well in a 96-well plate pretreated with 1% (w/v) agarose gel and cultured for 7 days. The tumor spheroids were incubated with the FAM-encapsulated liposomes for 4 hours. Subsequently, the spheroids were rinsed with PBS for three times, and subjected to confocal microscopy (TCS-SP8, Leica, Germany). Confocal slices were taken every 20 µm from the base to top of the spheroids.

### Cytotoxicity and apoptosis assay

The antitumor activity of HNK, DSF/Cu, free combo drugs, LIPO, and CDX-LIPO was determined by a standard MTT assay in U87 and C6 glioma cells. In brief, the cells were seeded in a 96-well plate with 5×10^3^ cells per well and cultured for 24 hours, and then treated with various drugs for 24 hours. The MTT reagent (5 mg/mL, 20 µL) was added to each well and incubated for another 4 hours. After removal of the medium, 200 µL of DMSO was added to each well and measured by a microplate reader at 490 nm (Multiskan, Thermo Fisher Scientific, USA). IC_50_ value based on DSF/Cu concentration was calculated using GraphPad Prism 8.

The U87 cells were plated onto the 6-well plates with 4×10^5^ cells per well 24 hours before treatment. The cells were treated with fresh culture medium containing various drugs (HNK, DSF/Cu, HNK+DSF/Cu, LIPO, or CDX-LIPO). After 24 hours, the cells were harvested and stained with a FITC-Annexin V apoptosis detection kit (Becton Dickinson, USA) following the manufacturer’s protocol, and measured by flow cytometry (CytoFLEX, Beckman Coulter, USA).

### Intracellular ROS detection

Intracellular ROS detection was performed using a reactive oxygen detection kit (Beyotime, China) by flow cytometry. The cells were seeded in the 6-well plates with 4×10^5^ cells per well and cultured for 24 hours. The cells were then treated with HNK, DSF/Cu, free combo drugs, LIPO, and CDX-LIPO for 12 hours. After wash, the cells were collected for ROS measurement.

### ICD induction study

The surface-exposure of CRT was assessed by immunofluorescence and flow cytometry. For immunofluorescence analysis, the C6 cells were seeded into the 12-well chamber slides at a density of 1×10^5^ cells/well and cultured for 6 hours. The cells were treated with various drugs for 6 hour, and then washed twice with PBS and incubated with anti-calreticulin antibody (Abcam, UK) for overnight at 4°C. On staining with DAPI, the cells were observed using a confocal fluorescence microscope.

For flow cytometry analysis, the C6 cells were treated as the method above, followed by incubation with the anti-calreticulin antibody for 1 hour. The cells were analyzed using flow cytometry.

The extracellular released ATP was examined using a chemiluminescence ATP determination ELISA kit (Life Technologies, USA). After incubation with various drugs for additional 6 hours, the C6 cell supernatant was collected and detected according to the manufacturer’s protocols.

### In vitro induction of antigen presentation

After treatment with the liposomes, the C6 cells were incubated with the BMDCs or BMDMs. The matured DCs (CD86^+^MHCII^+^) and BMDMs (CD80^+^/CD169^+^) were determined by flow cytometry.

### Effect of ICD on T cells

The BMDCs were primed with the C6 glioma cells that were pretreated with the drugs for 24 hours. The responder T cells isolated from the spleens of the Balb/c mice using Lymphocyte Separation Medium (Dakewe Biotech, China) were cocultured with the primed BMDCs at a ratio of 5/1 (T/BMDC) for 3 days. The IFN-γ^+^ T cells were collected and measured by flow cytometry.

### Intracranial glioma tumor models

The orthotopic glioma model was established by intracranially injecting the glioma cells into the right striatum using a micro-syringe needle (Hamilton, USA) at a volume of 5 µL for 4 min, with the following coordinate: 2.0 mm anterior, 2.0 mm lateral, and 3.0 mm deep. Due to their different properties of cell proliferation and growth characteristics, different cell concentrations (5×10^5^ C6 cells for a female Balb/c mouse and 5×10^6^ U87 cells for a female Balb/c nude mouse) were used for injection to establish the orthotopic glioma models.

### Biodistribution

The DiD-loaded liposomes were administered intravenously into the intracranial glioma-bearing mice. The mice were recorded by an IVIS imaging system (Caliper PerkinElmer, Hopkinton, Massachusetts, USA) at 1, 2, 4, 8, 12, and 24 hours. At 24 hours post-injection, the mice were euthanized and their heart, lungs, spleen, liver, brain, and kidneys were harvested. The fluorescent signal of each organ was measured by an IVIS imaging system. The brains were frozen in a Tissue-Tek OCT Compound and cut into the 10 µm thick slices (CM1950, Leica, Germany). The slices were stained with DAPI and observed by a confocal laser scanning microscope (TCS-SP8, Leica, Germany).

### In vivo treatment study

The C6 and U87 glioma orthotopic models were established according to the method above. Five days after implantation, the mice were randomly divided into six groups (n=10). PBS, HNK, DSF/Cu, DSF/Cu+HNK, LIPO, and CDX-LIPO were injected via tail vein every 2 days for five times (at a dose of DSF/Cu 1.5 mg/kg and HNK 15 mg/kg for liposomes), respectively. The liposomes were suspended in saline. The dosage regimen was based on a pilot study. It should be noted that due to the solubility problem, for the free drug groups, a dose of HNK was reduced to 3 mg/kg. The vehicle for free drugs consisted of 2.5% mixture of Cremophor EL and ethanol in 5% dextrose solution. The survival time of the mice in different groups was recorded to evaluate the anti-glioma therapeutic efficacy. The body weight of the treated mice was monitored. The mice were sacrificed when displayed signs of neurological deficits, and at the experimental endpoint the major organs were collected for further assay.

Organ coefficient was calculated using the following formula to preliminarily evaluate the side toxicity in the organs.

Organ coefficient (%)=Weight of an organ/Body weight × 100%.

### Flow cytometry

The organs were harvested from the glioma-bearing mice after treatment. The brain and spleen were homogenized in DMEM media containing collagenase IV and hyaluronidase (1 mg/mL) at 37°C. The tumor-infiltrating immune cells were collected by centrifugation (3500 rpm, 10 min), and resuspended in PBS containing 2% FBS and non-specific antibody binding was blocked with CD16/CD32. M1Φ was labeled with CD45, F4/80, and CD80 antibodies, and M2Φ labeled with CD45, F4/80, and CD206 antibodies. Activated NK cells were labeled with CD49b and NK1.1 antibodies and MDSC labeled with GR-1 and CD11b antibodies, respectively. Activated T cells were labeled with CD45, CD3, and CD4 or CD8 antibodies. Intracellular granzyme B and IFN-γ were stained using an intracellular staining kit (BD Biosciences, USA) following the manufacturer’s protocol. The cells were measured with a flow cytometer (CytoFLEX, Beckman Coulter, USA) and analyzed using CytExpert and FlowJo software.

### Lactic acid measurement

The tumor tissues of the mice after treatment as described above were harvested. The lactic acid level in the tumors was detected by a lactic acid assay kit (Nanjing Jiancheng Bioengineering, China).

### Cytokine measurement

ELISA was used to quantify the concentrations of the cytokines of IL-6 and TNF-α using the ELISA kits (Dakewe Biotech, China).

### Western blot analysis

The cells were lysed on ice for 10 min using a radioimmunoprecipitation assay (RIPA) buffer containing protease and phosphatase inhibitors (Beyotime, China). Similarly, the tumor tissues were also treated with a RIPA lysate buffer. The supernatants were obtained after centrifugation at a speed of 12,000×*g* for 20 min. The protein concentration was determined by using a BCA protein assay kit (Beyotime, China).

The samples were separated by SDS-PAGE gels and then transferred onto nitrocellulose membranes. After blocking with 5% bovine serum albumin (BSA) in Tris-buffered saline containing 0.1% Tween-20 for 1 hour at room temperature, the membranes were incubated with the specific antibodies overnight at 4°C. After wash for five times with TBST (each for 10 min), the membranes were incubated with HRP-linked secondary antibodies for 50 min at room temperature. After thorough washing, the proteins were visualized using ECL Western Blotting Substrate (Pierce, Thermo Fisher Scientific, USA) and detection was performed using a gel imaging analysis system (Bio-Rad, USA).

### Statistical analysis

All animal studies were performed after randomization. Data were analyzed by one or two-way analysis of variance with GraphPad Prism 8. All values are reported as means±SD with the indicated sample size, otherwise n=3. Multiple asterisks represent the statistical significance as *p<0.05, **p<0.01, ***p<0.001, and ****p<0.0001.
