# Supplementary material for: Remodeling tumor immune microenvironment (TIME) for glioma therapy using multi-targeting liposomal codelivery
Source: J Immunother Cancer. 2020 Aug 17;8(2):e000207. doi: 10.1136/jitc-2019-000207 (PMC7437977; doi:10.1136/jitc-2019-000207)
Supplement: Supplementary data [file jitc-2019-000207supp001.pdf]

Supporting Information

Remodeling Tumor Immune Microenvironment (TIME) for Glioma Therapy Using Multi-Targeting Liposomal Codelivery

*Zening Zheng [1,2,3], Jiaxin Zhang [2,4], Jizong Jiang [2], Yang He [2,5], Wenyuan Zhang [2,5], Xiaopeng Mo [2], Xuejia Kang [2], Qin Xu [1], Bing Wang [2], Yongzhuo Huang [2,5,6]**

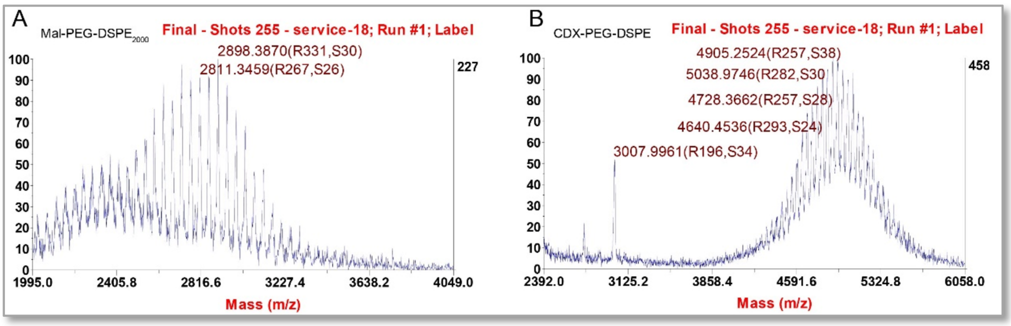

Figure S1 MALDI-TOF-MS assay of CDX-PEG_{2000}-DSPE.

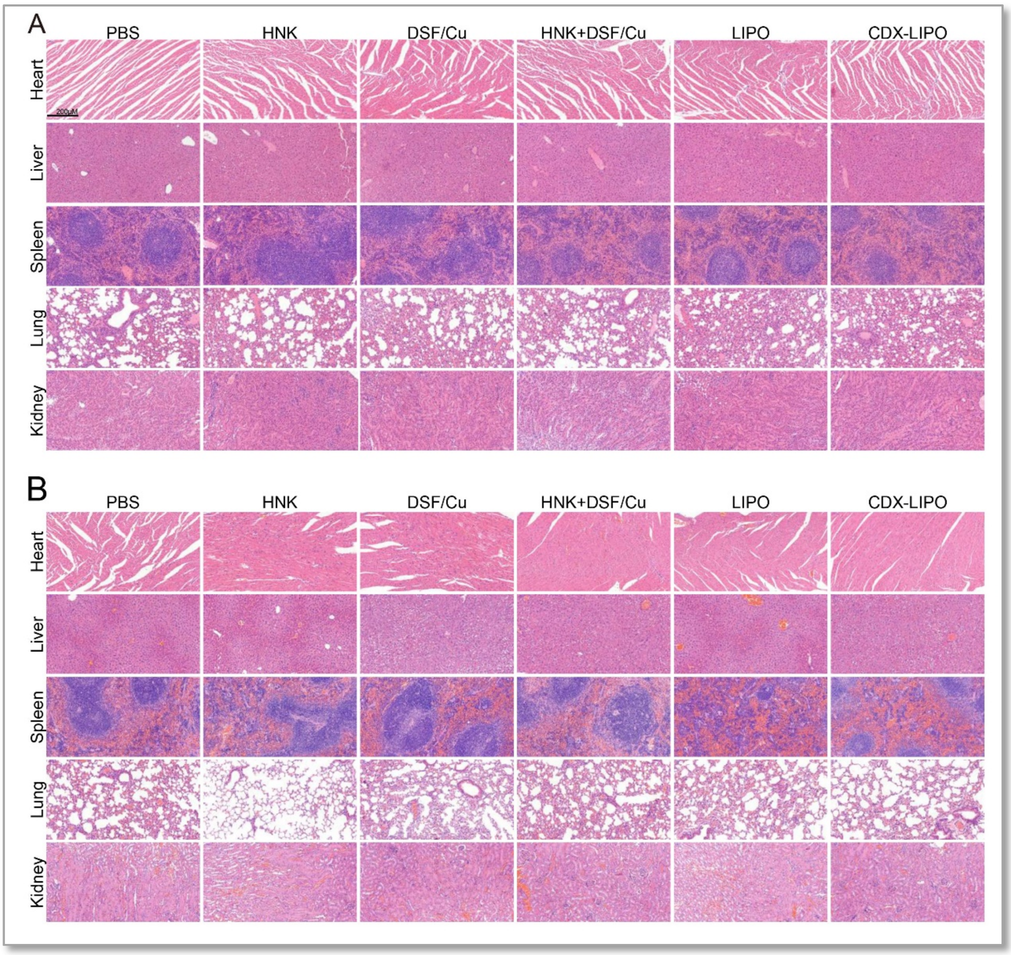

Figure S2 Histological examination of the major organs of the nude mice bearing orthotopic U87 glioma (A) and the Balb/c mice bearing orthotopic C6 glioma (B).

Table S1 Characterization of LIPO and CDX-LIPO.

	LIPO	CDX-LIPO
**Particle mean size (nm)**	118.7 ± 2.35	122.5 ± 3.42
**PDI**	0.22 ± 0.02	0.16 ± 0.01
**Zeta potential (mV)**	- 6.17 ± 0.21	1.36 ± 0.14

Note: Data are presented as mean ± SD (n = 3).

Table S2 Cytotoxicity of DSF/Cu and HNK with different combination ratios and the combination index (CI) values in the glioma cells.

	**DSF/Cu**	**HNK**	**DSF/Cu** (HNK 1 μg/mL)	**DSF/Cu** (HNK 2 μg/mL)	**DSF/Cu** (HNK 4 μg/mL)	**DSF/Cu +** (HNK 8 μg/mL)
**U87 IC_{50}** (μg/mL)	0.56	20.1	0.31	0.24	0.26	0.29
**CI Value**			0.95	0.72	0.81	0.89
**C6 IC_{50}** (μg/mL)	0.27	13.3	0.27	0.20	0.21	0.24
**CI Value**			0.99	0.74	0.77	0.86

Note: Data are presented as mean ± SD (n = 3). CI: 0.9-1.1: additive effect; 0.8-0.9: slight synergism; 0.6-0.8: moderate synergism; 0.4-0.6: synergism; 0.2-0.4: strong synergism.

Table S3 IC_{50} values in the U87 cells and C6 cells from Figure 3A, B.

**IC_{50}（μg/mL）**	**HNK**	**DSF/Cu**	**DSF/Cu + HNK**	**LIPO**	**CDX-LIPO**
**U87**	>2.56	0.56	0.38	0.26	0.16
**C6**	>2.56	0.28	0.22	0.15	0.11

Table S4 Body weight changes after treatment.

C6-glioma bearing mice	PBS	HNK	DSF/Cu	CO	LIPO	CDX-LIPO
**Mean ± SD**	16.37 ± 2.60	16.19 ± 2.06	16.20± 1.62	16.14 ± 1.89	15.28 ± 1.91	18.22 ± 1.44
**Coefficient of variation**	15.81%	12.70%	9.98%	11.71%	12.53%	7.88%

U87-glioma bearing mice	PBS	HNK	DSF/Cu	CO	LIPO	CDX-LIPO
**Mean ± SD**	16.76 ± 1.08	15.97 ± 2.28	16.36 ± 2.01	16.58 ± 1.55	15.08 ± 1.38	16.27 ± 1.14
**Coefficient of variation**	6.43%	14.29%	12.31%	9.37%	9.13%	7.03%

Note: The body weight variations were represented by means ± SD and coefficient of variation (CV), (n =10). There was no significant difference of the above parameters between the treatment groups and the control group ($p > 0.05$).
